# Informatics and Health

**Published:** 2014-06-25

**Authors:** VL Purcarea

**Affiliations:** „Carol Davila” University of Medicine and Pharmacy Bucharest, Romania

The depth of the changing process in health, its dimension and dynamics do not depend only on the political will and the punctual subjective aspirations, but especially on the existence and sufficiency of the necessary objective conditions, on the first place being the managerial ones. The special role of the manager in the context of the problems in the nowadays health system is imposed by the necessity of creating a general capacity of innovation, flexibility, stability, assuring the success even in special situations. In this context, the theoretical but especially the methodological approach of the management of the hospital informational system has special practical valences. This is what distinguished Prof. Florian Popa, MD, Vice-president of the Health Commission of the Senate of Romania and President of the National Council of Attesting the Titles, Diplomas and University Certificates in Medicine, affirms in the preface of a book distinguished with “Victor Babes Prize” by the Romanian Academy, inviting the readers not only to reflections and dialogue but also to a rapid effort of implementing or completing the information implementation processes, the new reality in health imposing this, rigorously and impetuously. 

 This undoubtedly imposes thorough knowledge in the domains of the distributed leading processes, medical units and management of the resources of these units (applying concepts and methods specific to Artificial Intelligence) and an unmediated connection to the informational society. 

 However, the knowledge society represents more than the informational society, the first being possible only grafted on the informational society, and cannot be separated from it because of the major role which belongs to the information, meaning knowledge in society. 

 Globalization, the development of the services and especially the exponential raise of the needs and desires of the patients impose the abandonment of the organization model centered on internal problems and the adaptation to other models, centered on the patients’ needs by the institutions in the medical system. This new wave of dynamic relationships and innovation, centered on the patient needs the integration at the overall level of the processes, applications and systems at an unprecedented level, with benefic results due to the use of technology at a large scale. As a consequence, what is vital in this context, is the use of Information and Communications Technology as a support for the activities of healthcare specific to the persons, the development of the conceptual-theoretical and methodological frame of Information and Communications Technology and the modeling and development of complex informatics systems specific to medicine, generating new instruments and technologies for specific applications, while mentioning the thematic areas such as telecare, telehealth, eHealth, assistive technology, ambient assisted living in smart houses, preparing competent specialists in the fundamental research with interdisciplinary applications and not least, raising the visibility of the Romanian research in the fields of complex informatics systems at the international level.

**Fig. 1:**
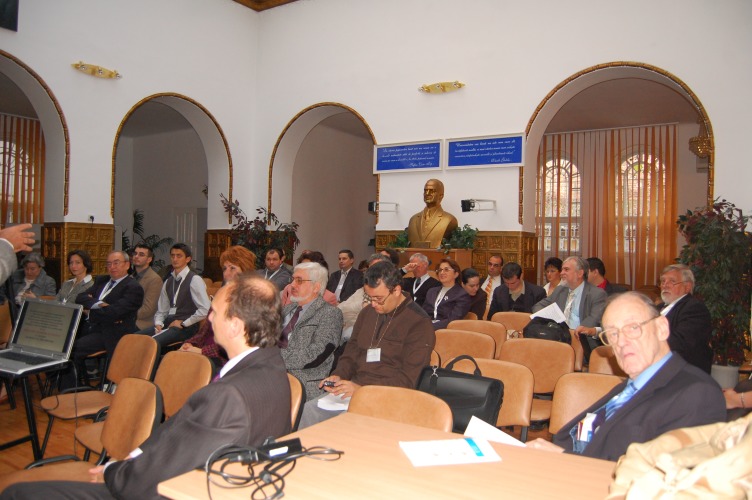
The conference in Timisoara

**Fig. 2:**
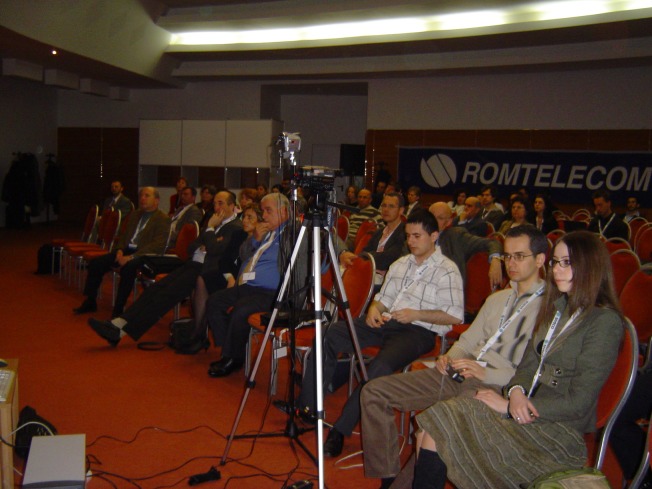
The conference in Arad

Important steps were taken and are still being taken in most of the university centers in Romania, with a subjective mention, for the Automatics and Computers Faculty of the Polytechnic University in Bucharest. We are saluting the actions taken in this direction by the Romanian Society of Medical Informatics, which, involves more and more in this devotedly, with professionalism and more efficiently. 

**Fig. 3:**
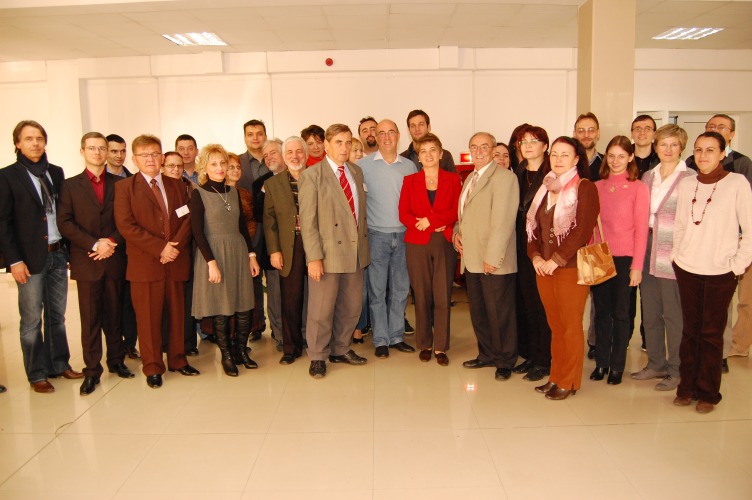
Participants in the Conference

**Fig. 4:**
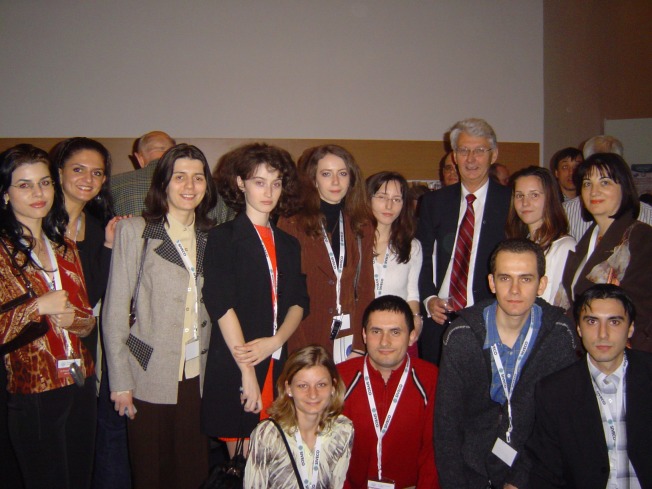
Group picture of the participants in the Conference

That is why, it is our great pleasure to announce that, this year, RoMedinf Annual Conference of the Romanian Society of Medical Informatics will be held on more working groups - Interoperability and HER – (Health Electronic Registration, Education in Biomedical Informatics, Medical Imagistics, Dental Informatics, Decisional Systems and Medical Databases, etc.) in the great and full of signification halls of “Carol Davila” University of Medicine and Pharmacy in Bucharest. 

**Fig. 5:**
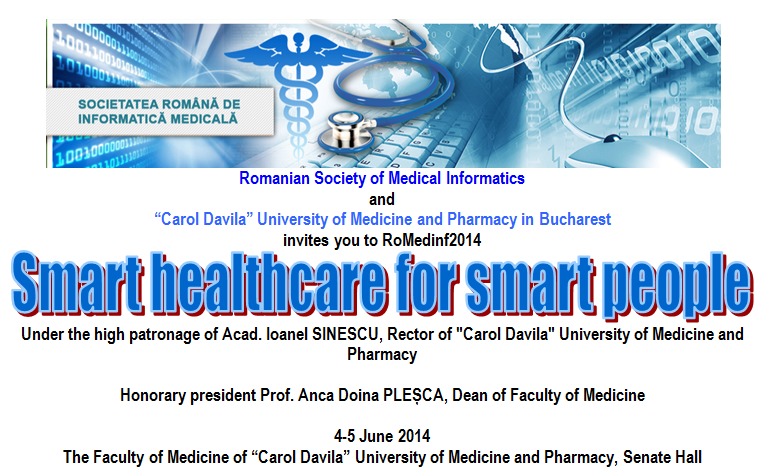
The banner representing RoMedinf 2014 event

Obviously, it is the expression of not only the seriousness and the implication of the specialists in the field of medical informatics, in finding a faster and more performing solution from the qualitative point of view of the healthcare needs, but also the responsiveness to the new ideas, to interdisciplinarity and performance of the oldest school of medicine in Romania. 

**Executive Editor**

**Assoc. Prof. Dr. Eng. Victor Purcarea**

